# Doppler velocimetry of the middle cerebral artery and basilar artery in clinically healthy dogs of different age groups

**DOI:** 10.1186/s12917-025-04644-9

**Published:** 2025-03-19

**Authors:** Tamiris Disselli, Denise Jaques Ramos, Luiz Paulo Nogueira Aires, Diego Rodrigues Gomes, Diana Villa Verde Salazar, Stéfany Tagliatela Tinto, Ricardo Andres Ramirez Uscategui, Igor Cezar Kniphoff da Cruz, Marcus Antônio Rossi Feliciano

**Affiliations:** 1 Department of Veterinary Medicine, Faculdade de Zootecnia e Engenharia de Alimentos, Universidade de São Paulo, Rua Duque de Caxias, 225 - Jardim Elite, Pirassununga, São Paulo, 13635-000 Brazil; 2 Department of Veterinary Surgery, Faculdade de Ciências Agrárias e Veterinárias, Universidade Estadual Paulista, Rua Prof. Paulo Donato Castellane, s/n - Vila Industrial, Jaboticabal, São Paulo, 14884-900 Brazil; 3 Department of Animal Health, Faculdade de Medicina Veterinária e Zootecnia, University of Tolima, Barrio Santa Helena Parte Alta, Ibague, Tolima 730006299 Colombia; 4 Department of Large Animals Clinic, Universidade Federal de Santa Maria, Hospital Veterinário Universitário, Av. Roraima, 1000, prédio 97, Santa Maria, Rio Grande do Sul 97105-900 Brazil

**Keywords:** Brain, Canine, Hemodynamic indexes, Transcranial ultrasound

## Abstract

**Background:**

Transcranial Doppler ultrasound is a low-cost test that provides real-time information on brain hemodynamics and makes it possible to detect and monitor hemodynamic disorders non-invasively by calculating Doppler velocimetric values. In veterinary literature, studies related to this diagnostic tool are still scarce, with most dating back more than a decade and very few involving the Doppler study of the arterial circle. Therefore, the aim of this study was to evaluate qualitatively (color Doppler and spectral classification) and quantitatively (pulsed Doppler) the middle cerebral artery (MCA) and basilar artery (BA) of clinically healthy dogs of different age groups to determine normal hemodynamic values and their relationship with the age of the animals.

**Results:**

The end-diastolic velocity (EDV) and peak systolic velocity (PSV) values differed between young, adult, and elderly dogs for the right MCA and BA, and the left MCA, respectively (*p* < 0.05). No differences were observed in the resistivity and pulsatility indices between the three age groups.

**Conclusions:**

Doppler velocimetric flow velocities of right and left middle cerebral artery (RMCA and LMCA) and basilar artery (BA) vary with advancing age in clinically healthy dogs. Therefore, care must be taken while evaluating cerebral hemodynamic indexes in normal as well as diseased dogs belonging to different age groups.

## Background

Transcranial ultrasound (TUS) has the advantage of being a non-invasive diagnostic tool of important diagnostic value, due to its speed, low cost and ready availability, without the need for patient sedation, allowing serial examinations to be carried out over time [1]. In addition, it can complement the imaging findings of other imaging methods, such as computed tomography (CT) and magnetic resonance imaging (MRI) with good accuracy when compared to these modalities [2], and is often considered superior to CT such as in the differentiation of tumor components [3] and the assessment of lesions in brain tissue and intravascular flow [4].

Transcranial Doppler ultrasound (TCDUS) allows rapid, non-invasive, real-time measurement of cerebrovascular function [5] by providing color images of intracranial and basilar arterial flow [6], especially the rostral and middle cerebral arteries [7], complementing B-mode.

In veterinary medicine, studies related to this imaging modality are limited and old [8], with most of them using a limited and heterogeneous patient population, post-mortem evaluations, as well as sedated or anesthetized dogs [9–15]. Such situations can cause variations in the hemodynamic indexes acquired via transcranial Doppler ultrasonography, making it impossible to establish reliable normal values for the canine species. In addition, most studies that have described normal values have not analyzed all the Doppler indexes or standardized the animals in terms of age, weight or size [8–10, 12–14, 16, 17], further limiting the stipulation of reference values that can be used in the practical clinical context.

Since the number of dogs with neurological disorders in the small animal clinic has increased in recent decades, as well as the life expectancy of these animals, a better knowledge of encephalic vascular behavior at different stages of life is necessary to understand the effect of age on the cerebral hemodynamic indexes [9, 18]. Studies on transcranial Doppler ultrasonography derived hemodynamic indexes of the cerebral arteries with respect to age, body weight and size in clinically healthy dogs have not been reported till date.

The use of a more homogeneous sample of animals, standardizing age, weight and size, will allow reliable and reproducible normality values to be obtained, contributing to a more precise and individualized diagnosis and monitoring of encephalic hemodynamic disorders in this species.The aim of this study was to evaluate and establish the main Doppler velocimetric values of normality for the middle cerebral artery and basilar artery in clinically healthy young, adult and elderly dogs, using TCDUS as a diagnostic tool.

## Results

A total of sixty-four dogs were evaluated out of which six dogs were excluded owing to thrombocytopenia in three dogs, thrombocytosis in one dog and history of seizures in other two dogs.

Of the fifty-eight dogs evaluated, thirty-three (56.9%) were females and twenty-five (43.1%) males. Regarding the breeds, the most represented were Shih Tzu (12 dogs) and Yorkshire Terrier (14 dogs). The average body weight was 5.03 ± 1.75 kg, with significant differences observed between the age groups (*p* < 0.05) (Table 1).

**Table 1:** Frequency of distribution of breed, age, gender, and body weight in a transcranial pulsed doppler examination of 58 clinically healthy dogs grouped by age (young, adult, elderly)

Parameter	Total	Young dogs	Adult dogs	Elderly dogs
**Total Number of Dogs**	58 (100%)	10 (17.2%)	38 (65.5%)	10 (17.2%)
**Gender Distribution**				
Female	33 (56,9%)	5 (8.62%)	22 (37.9%)	6 (10.4%)
Male	25 (43.1%)	5 (8.62%)	16 (27.6%)	4 (6.89%)
**Breeds**				
Spitz	2 (3.44%)	Mongrel	10 (17.2%)	
Maltese	3 (5.17%)	Pinscher	10 (17.2%)	
Poodle	6 (10.3%)	Shih Tzu	12 (20.7%)	
Yorkshire Terrier	14 (24.1%)			
**Age (Mean ± SD) years**	5.7 ± 3.7	0.5 ± 3.4*	5.6 ± 2.3*	11.3 ± 1.3*
**Body weight (Mean ± SD) kg**	5.0 ± 1.8	3.4 ± 0.9*	4.9 ± 2.0	6.8 ± 2.4*

*Values were considered significant in an ANOVA test and Tukey Pos-test when *P* < 0.05

All dogs included in the study had normal B-mode transcranial ultrasound scans, with no focal, diffuse or structural lesions, except for the examination of two Shih Tzu dogs, eleven years old, and a Poodle, thirteen years old, in which there was a sonographic image of diffuse blurring of the cerebral sulci. However, this finding was attributed to senility, since none of the animals had a history or clinical signs compatible with neurological alterations, such as cognitive dysfunction, for example, which could justify this sonographic finding. The total time taken to carry out the study protocol from anamnesis to ultrasound and laboratory tests, varied between 1 h and 1 h 30 min, respectively depending on the patient’s collaboration. The average time taken to perform the transcranial ultrasound (TCUS) technique was 20 to 30 min.

**Fig. 1:**
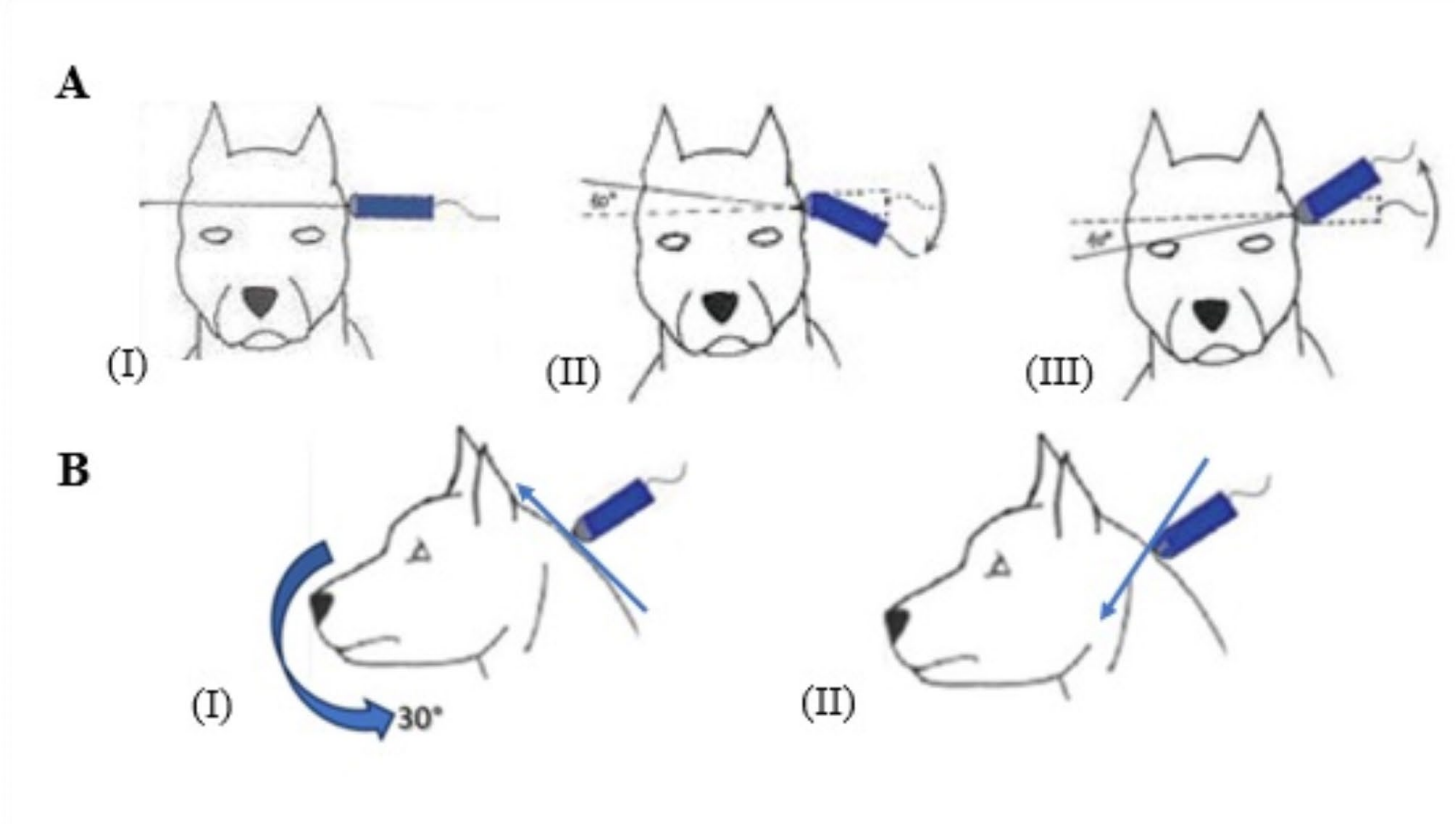
Schematic drawing illustrating the transducer positioning and maneuvers for transcranial ultrasound scanning. (**A**) Transtemporal window transducer perpendicular to the temporal bone with median dorsal plane (I), cranial (II) and caudal (III) oblique dorsals. Dashed lines represent “fan” scan. (**B**) Suboccipital window: transducer perpendicular to the foramen magnum in (I) longitudinal planes, with a wide arrow descending cervical ventroflexion of 30º, and transverse (II). Thin arrows indicate the direction of the cranial marking of the transducer. Source: Adapted from (Cintra et al., 2014) [19] and (Carvalho,2021) [20]. Adjustments to **B** (I and II) to include new visual elements

**Fig. 2:**
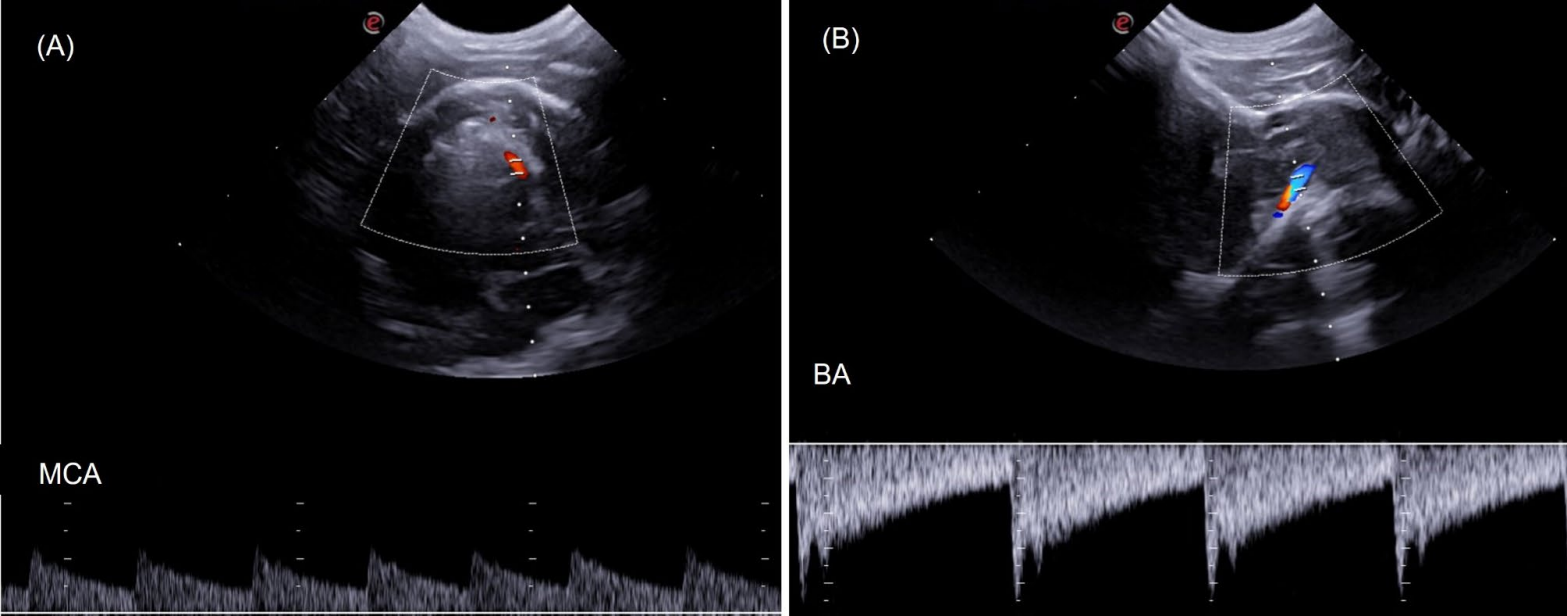
Transcranial sonographic images from the Triplex Doppler study of the cerebral and basilar arteries of clinically healthy canines. (**A**) Middle cerebral artery (MCA), transtemporal window. Doppler adjustments Color Doppler gain = 66% and PRF = 4.3 kHz., (**B**) Basilar artery (BA), suboccipital window; Color Doppler gain = 60%, PRF = 1.9 KHz

In a qualitative analysis, the results of TCUS showed increased visualization of B-mode intracranial structures and color Doppler mapping of the basilar artery (Fig. 1) in miniature dogs compared to small dogs and brachycephalic dogs. The visibility of middle cerebral arteries and basilar artery on color Doppler was correlated with age, sex (males and females) and the sides of cerebral hemisphere assessed (left and right). A statistical difference was observed in the visibility of basilar artery between young and old dogs (Fig. 2) and between male and female dogs, being more easily visible in young and male dogs compared to old and female dogs.

No significant differences were observed in relation to the sex of animal (Table 2) and sides of cerebral hemispheres evaluated (Table 3). In a quantitative analysis, the values of hemodynamic indexes for the arteries evaluated were acquired for each age group. Quantitative data revealed a negative correlation between cerebral artery flow velocities and the age of the animals. The correlations observed were as follows: PSV of the left MCA (*r* = -0.412) and right MCA (*r* = -0.402), EDV of the left MCA (*r* = -0.461) and right MCA (*r* = -0.451), PSV of the BA (*r* = -0.43), and EDV of the BA (*r* = -0.49). Furthermore, significant differences were identified in some velocities across age groups: EDV of the right MCA (*p* = 0.0339) and BA (*p* = 0.015), as well as PSV of the left MCA (*p* = 0.049), with a 95% confidence interval (Table 4).

**Table 2:** Mean ± standard deviation (SD) of the hemodynamic indexes of the middle cerebral arteries (right and left) and Basilar arteries compared between sex. Data obtained in a transcranial pulsed doppler examination of 58 clinically healthy dogs grouped by age (young, adult, elderly)

Arteries		RMCA		LMCA		BA	
Indexes	Gender	Mean ± SD	*P* value	Mean ± SD	*P* value	Mean ± SD	*P* value
PI	Male	1.09 ± 0.29	0.761	1.17 ± 0.24	0.192	1.72 ± 0.47	0.924
	Female	1.06 ± 0.27		1.07 ± 0.22		1.73 ± 0.47	
RI	Male	0.64 ± 0.10	0.802	0.64 ± 0.08	0.210	0.75 ± 0.08	0.461
	Female	0.63 ± 0.10		0.67 ± 0.07		0.76 ± 0.75	
PSV (cm/s)	Male	27.4 ± 12.0	0.272	24.5 ± 9.09	0.424	52.4 ± 19.5	0.423
	Female	31.4 ± 9.91		26.6 ± 7.72		56.9 ± 21.5	
EDV (cm/s)	Male	10.1 ± 5.07	0.382	9.24 ± 3.38	0.785	13.4 ± 6.48	0.763
	Female	11.6 ± 4.85		8.93 ± 3.59		13.9 ± 6.73	

*Values were considered significant in a Student’s t-test when *P* < 0.05. RMCA: right middle cerebral artery; LMCA: left middle cerebral artery: BA: basilar artery; PI: pulsatility index; RI: resistivity index; PSV: peak systolic velocity; EDV: end-diastolic velocity

**Table 3:** Mean ± standard deviation (SD) of the hemodynamic indexes of the middle cerebral arteries compared to the sides of the hemisphere evaluated (right or left). Data obtained in a transcranial pulsed doppler examination of 58 clinically healthy dogs grouped by age (young, adult, elderly)

Arteries	RMCA	LMCA	
Indexes	Mean ± SD	Mean ± SD	*P* value
PI	1.10 ± 0.28	1.10 ± 0.23	0.960
RI	0.63 ± 0.09	0.66 ± 0.07	0.463
PSV (cm/s)	30.0 ± 11.0	26.0 ± 8.30	0.252
EDV (cm/s)	11.0 ± 4.90	9.10 ± 3.50	0.225

Values were considered significant in an Student’s t-test when *P* < 0.05. RMCA: right middle cerebral artery; LMCA: left middle cerebral artery: PI: pulsatility index; RI: resistivity index; PSV: peak systolic velocity; EDV: end-diastolic velocity

**Table 4:** Mean ± standard deviation (SD) and confidence interval at 95% of significance of hemodynamic index values of the middle (right and left) and Basilar cerebral arteries compared by age group (young, adult and elderly). Data obtained in a transcranial pulsed doppler examination of 58 clinically healthy dog

Arteries		RMCA					LMCA					BA				
Indexes		Young	Adult	Elderly	*P* value		Young	Adult	Elderly	*P* value		Young	Adult	Elderly	*P* value	
PI		0.86 ± 0.19 ^A^	1.10 ± 0.28 ^A^	1.06 ± 0.24 ^A^	0.079		1.17 ± 0.21 ^A^	1.10 ± 0.26 ^A^	1.18 ± 0.15 ^A^	0.725		1.43 ± 0.42 ^A^	1.80 ± 0.46 ^A^	1.94 ± 0.44 ^A^	0.054	
		0.65 - 1.10	0.94 - 1.30	0.74 - 1.40			0.89 - 1.40	1.00 - 1.20	0.93 - 1.40			1.00 - 1.70	1.50 - 1.90	1.30 - 2.70		
RI		0.56 ± 0.09 ^A^	0.65 ± 0.09 ^A^	0.62 ± 0.09 ^A^	0.115		0.66 ± 0.07 ^A^	0.65 ± 0.08 ^A^	0.68 ± 0.06 ^A^	0.778		0.71 ± 0.09 ^A^	0.72 ± 0.08 ^A^	0.80 ± 0.09 ^A^	0.107	
		0.47 - 0.71	0.58 - 0.71	0.52 - 0.75			0.57 - 0.76	0.61 - 0.69	0.59 - 0.77			0.64 - 0.79	0.73 - 0.78	0.69 - 0.92		
PSV (cm/s)		34.7 ± 16.5 ^A^	30.0 ± 9.60 ^A^	20.8 ± 5.71 ^A^	0.101		27.7 ± 9.36^A^	27.0 ± 7.80 ^AB^	18.2 ± 6.21^BC^	0.049*		63.4 ± 19.9 ^A^	55.0 ± 21.0 ^A^	42.2± 15.2 ^A^	0.112	
		11.0 - 53.0	24.0 - 36.0	17.0 - 31.0			20.0 - 40.0	21.0– 32.0	11.0– 26.0			41.0 - 83.0	43.0 - 60	26.0 - 67.0		
EDV (cm/s)		15.1 ± 7.45 ^A^	11.0 ± 4.10 ^AB^	9.00 ± 6.14 ^BC^	0.034*		9.45 ± 3.18 ^A^	9.51 ± 3.55 ^A^	5.98 ± 1.52 ^A^	0.103		18.1 ± 4.23^A^	14.0 ± 6.60 ^AB^	9.00 ± 6.14 ^BC^	0.015*	
		5.80 - 23.0	7.40 - 12.0	5.00 - 11.0			6.68– 12.2	8.25 - 10.8	2.95– 9.01			15.0 - 23.0	10.0 - 17.0	3.00 - 21.0		

Values were considered significant in an ANOVA test and Tukey Pos-test when *P* < 0.05. RMCA: right middle cerebral artery; LMCA: left middle cerebral artery: BA: basilar artery; PI: pulsatility index; RI: resistivity index; PSV: peak systolic velocity; EDV: end-diastolic velocity

**Fig. 3:**
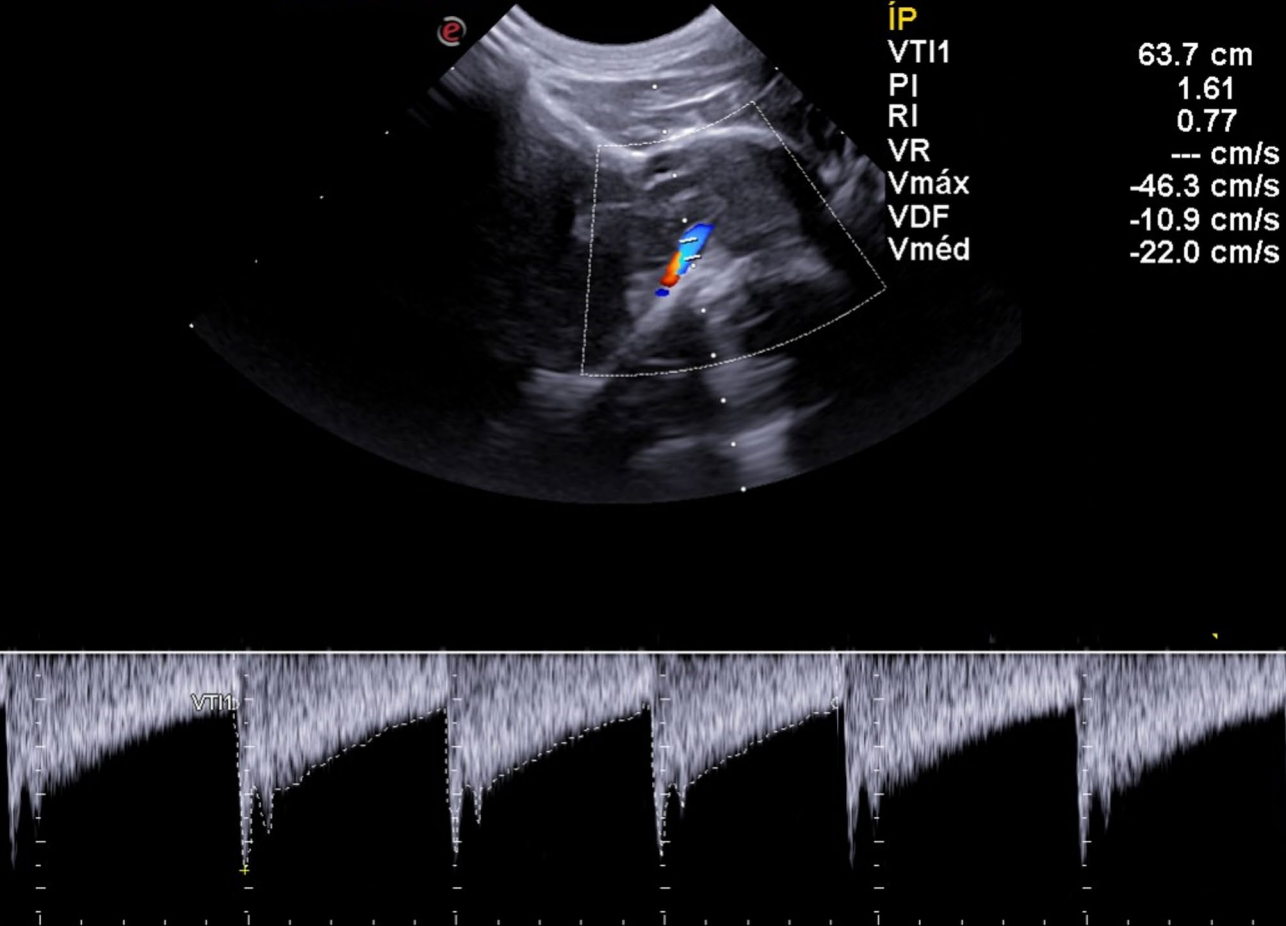
Sonographic image obtained in a transcranial examination (suboccipital window) of a Shih Tzu showing the basilar artery on color Doppler (flow in blue) and its corresponding pulsed spectrum, highlighting the measurement of three similar and consecutive waves (dotted line). In the upper right corner, values of hemodynamic indexes are described VTI1 = time velocity integral; PI = pulsatility index; RI = resistivity index; Vmax = maximum speed; EDV = end-diastolic velocity; Vmed = average speed. Doppler adjustments: Color Doppler Gain = 60%, PRF = 1.9 KHz

**Fig. 4:**
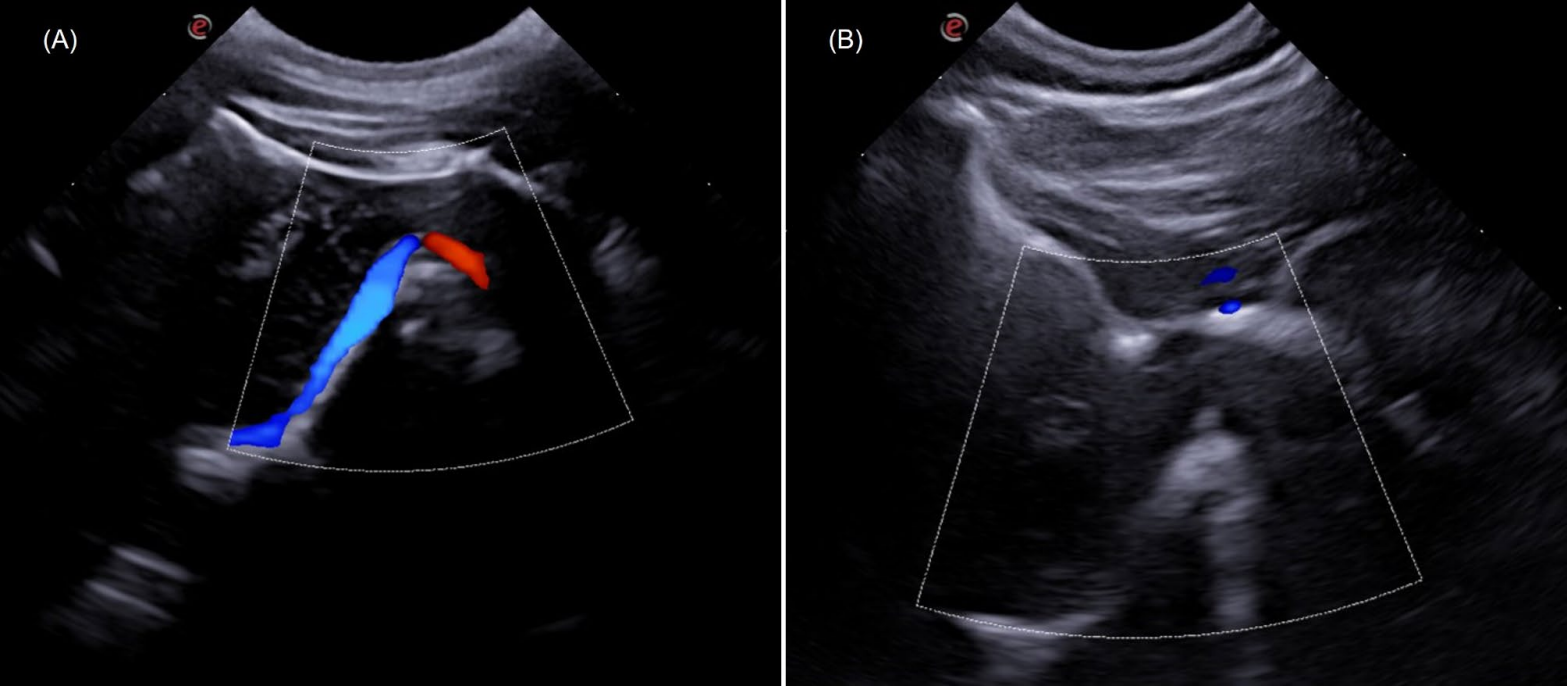
Transcranial sonographic images obtained in the suboccipital window (longitudinal plane) showing color Doppler mapping of the basilar artery of canines (**A**) pinscher, weighing 2.850 kg (**B**) Shih Tzu, weighing 9.4 kg. Note the difficulty in acquiring the color Doppler signal in brachycephalic dogs of larger size and body weight compared to mesaticephalic dogs of smaller weight and miniature size. Doppler adjustments (**A**) Gain = 60%; PRF = 4.3 kHz; (**B**) Gain = 60%, PRF = 2.9 KHz

Qualitative analyses of flow direction and wave spectrum morphology of right and left middle cerebral and basilar arteries showed no changes. A single-phase laminar flow with a low resistivity pattern and a high diastolic flow for the middle cerebral arteries (Fig. 3) and a single-phase flow with low pulsatility and resistance, indicated by broad, continuous systolic peaks, with the presence of occasional dicrotic peaks in some tracings for basilar artery was recorded (Fig. 4).

## Discussion

In general, it can be said that the studies currently available in the literature related to the assessment of cerebral hemodynamics in dogs leave many gaps in relation to the standardization of the animals, limiting the establishment of normal values. This study is unprecedented and enriching in bringing normal values for clinically healthy dogs of different age groups, with standardized weight and size.

In this study, the findings for B-mode TUS corroborate those other studies [19, 20], who found better quality for miniature dogs, with a direct relationship between the encephalic structures assessed and the weight and size of the animal. The same can be seen in the Doppler study, in which it was easier to map the arteries in miniature dogs than in larger dogs. The main factor described in the literature is the thickness of the skullcap, with smaller dogs having a thinner temporal region than larger dogs, making it easier for the sound beam to pass through and for the technique to be carried out properly [11, 20–22].

The transtemporal window was generally effective for both B-mode and Doppler evaluations, as its thinner bone provides a natural acoustic window, making it suitable for adult dogs after fontanelle closure. Although previous studies [23, 24] suggest that skull thickness and cranial conformation can impact ultrasonographic quality, in the present study, the transtemporal window did not limit either B-mode or Doppler evaluations. However, in brachycephalic dogs, the technique was more challenging due to their thicker skulls and breed-specific cranial conformation, which made it more difficult to access the brain structures, particularly during Doppler assessment. The suboccipital window was also more restricted in these dogs. Therefore, the application of the transtemporal window in dogs, especially across different age groups, remains underexplored, highlighting the need for further research to improve its reliability across breeds and life stages.

The results related to Doppler velocimetric parameters showed a significant influence of age on the flow velocities of the arteries evaluated (EDV of the right MCA and BA, and PSV of the left MCA), with a negative correlation and higher values for young dogs and lower values for elderly dogs [11, 20–22]. On the other hand, the same did not occur when analyzing the resistive indexes, with the results being similar between the age groups [11, 20–22].

Although studies specifically related to vein changes with ageing are considered rare in both human medicine [23] and veterinary medicine [24], this difference between the flow velocities observed in the extreme age groups corroborates research carried out in other animal species [25–30].

The differences in the behavior of the venous system in the brain of the elderly compared to the young are not yet well established in the animals’ species [25–30]. Research in humans suggests that advanced age is associated with brain atrophy [25–30], altered neural signaling [27] and deficiencies in aspects of cognition [31, 32] and that these changes appear to be related to thinning of the endothelium and reduction in capillary diameter [25. In humans, it is known that although cerebral compliance is maintained with the effect of age, there is a loss of pulsatility of the arterial impulse [25], establishing a negative correlation between total and cerebral blood flow and their reduction throughout the individual’s life [27], more evident at extreme ages [25], corroborating the results of this study for the canine species.

The significantly higher flow velocities observed in young dogs may be associated with greater vascular elasticity and lower peripheral resistance, consistent with patterns observed in studies on human neonates [18]. Conversely, the lower velocities in elderly dogs likely reflect reduced vascular compliance and cerebral perfusion, aligning with hypotheses proposed in previous research on mammals [25, 29].

In general, comparing the hemodynamic index values described for the MCA and BA in our study for the canine species with those reported in the literature is difficult, since the heterogeneity of the sample found in these works makes it impossible to establish a reliable correlation. In addition, there is no study describing Doppler velocimetry values for the different age groups, making comparative analysis of the results even more difficult. When comparing the parameters evaluated there was a significant difference between some flow velocities (EDV of the right MCA, PSV of the left MCA and EDV of the BA).

Given the above, we did not find a justification with a literary basis for this fact, as only two studies [13, 33] carried out the evaluation and comparison between the right and left MCA, but in none of them there was a comparison between animals of different age groups carried out. In human neonates, asymmetries in cerebral blood flow have been observed, suggesting that lateralization in vascular anatomy and perfusion could play a role in differential blood flow between the right and left hemispheres. Such asymmetries in blood flow, as seen in other species, may be influenced by a variety of factors, including age, vascular compliance, and the anatomical structure of the cerebral vasculature [34–36], which might explain some of the differences observed in our study. However, in the case of canines, where comparable data is scarce, these asymmetries, particularly across age groups, remain largely unexplored.

Comparing the values in this study with those in clinically healthy un-anaesthetized dogs [9, 10, 13, 14], the PI values for adult dogs were lower [13]. On the other hand, RI values were similar [9, 13].

Regarding MCA flow velocities, the PSV and EDV values for adult and elderly dogs differed from those described in the literature [13]. For young dogs, the values were lower than those reported in a study of neonate canines [9], but the researchers reported a subsequent decline in these velocities in the animals assessed at 3 and 4 weeks of age, matching our results for the youngest dogs that we evaluated. This fact has also been verified in humans, with a linear increase in flow velocities being reported in neonates up to 2 months of age, with a reduction to 70% of the maximum value at 16 years of age [18].

Concerning to BA, the studies currently available in literature involving its hemodynamic assessment in dogs are numerically superior to those that aimed to do the same assessment with cerebral arteries. This can be explained by the fact that the BA is easier to be seen [37], since it is considered the largest artery in the posterior territory [38] and it is located in a single trunk, running along the midline of the ventral surface of the medulla and pons [39], making it easy to see. In addition, its access through the suboccipital window does not interpose the skullcap, reducing the possibility of image artifacts, regardless of the animal’s size or cranial conformation. This was also seen in our study, in which the BA was visible in all 58 dogs evaluated, while the MCA was visible in only 40 animals.

About the evaluation of their indexes, we also found difference between EDV for young, adult and elderly dogs, with higher values ​​for young and lower elderly dogs. Most of the values for flow velocity were lower than those reported in the literature [13, 17], except for one study with which similarity was obtained [15]. The pulsatility index (PI) values in the present study for adult and elderly dogs were similar to those found in the literature [8, 13, 17], as were the resistivity index (RI) values for young [9], adult and elderly dogs [8, 13–15]. Saito et al. [11] described RI values higher than 0.68 as predictive of hydrocephalus, however, in the present study we obtained a mean RI of 0.76 ± 0.09 for animals without clinical signs or sonographic images suggestive of ventriculomegaly, which reiterates the importance of this study.

When analyzing the wave spectra of the arteries evaluated, it is known that the morphology of normal wave flow in the cerebral arteries shows a rapid systolic increase, reflecting normal proximal vessels and cardiac function, but the characteristics of the diastolic portion of the waveform are determined by the resistance of the vascularization of the distal bed. In this study, there were no changes in morphology and flow direction when compared to the literature [40]. For BA, the finding related to the presence of dicrotic peaks, verified in the evaluation of some clinically healthy animals in this study, can be considered a variation from normality, corroborating Carvalho et al. (2009) [33], who described this morphological pattern as a possible variation in the canine species.

Some limitations can be considered in this study, such as: small number of breeds used and possible racial variations in terms of hemodynamic values ​​and morphometry of the ventricular system. It would also be interesting to consider a larger number of animals, which was difficult in our study considering the use of owner dogs who agreed to participate. Although a liquor collection was not performed, the physical and hematological evaluations carried out did not show any findings suggestive of infectious changes in dogs, thus reducing a possible bias in this research. However, it would be pertinent to consider its implementation in future studies as well as complementary non-invasive tests (measuring blood pressure and electrocardiogram) to confirm the absence of cardiovascular diseases more effectively, since variations in pressure and the cardiac cycle can directly influence in brain hemodynamic values.

With these improvements, we believe that, although promising, the results of this study can be improved, reflecting more reliable and reproducible normal values.

## Conclusion

Doppler velocimetric flow velocities of right and left middle cerebral artery (RMCA and LMCA) and basilar artery (BA) vary with advancing age in clinically healthy dogs. Therefore, care must be taken while evaluating cerebral hemodynamic indexes in normal as well as diseased dogs belonging to different age groups.

## Methods

A total of 58 dogs (up to 10 kg) of various breeds, aged between 3 months and 14 years and weighing (1.5 to 10 kg) were used for the study. The animals were allocated into three different age groups: young dogs aged between 3 months and 1 year (*n* = 10); adult dogs aged between 1 and 10 years (*n* = 38) and elderly dogs aged between 10 and 14 years (*n* = 10). Animals under 3 months of age were not included in study, since higher hemodynamic values are expected, according to studies with human neonates [41], interfering with the final values. All the animals were from owners, remaining at the study site only during the procedures.

The animals’ health was certified through procedures carried out sequentially on the same day: These included an initial assessment protocol (anamnesis) to obtain the animals’ clinical history and morbid history, paying special attention to the neurological system; a physical examination to assess their general state of health; transcranial (B-mode and Doppler) and abdominal ultrasound (to assess their general state) and, finally, a laboratory test (complete blood count).

The blood count was chosen as the last step to minimize the effects of stress on the animal, since in human neonates there has been a direct association between alertness and increased blood flow velocity [18]. All acquired data was filed with the animals’ identification. Dogs with a history or clinical signs suggestive of neurological diseases, unruly or very agitated to the point of not allowing handling, with changes in transcranial ultrasound examination (presence of focal, diffuse or structural lesions), or with significant changes in laboratory examination were excluded.

B-mode and Doppler ultrasound evaluation of nervous system was performed by a sonographer with seven years of experience and previous training in performing the technique, in the following chronology: two-dimensional transcranial ultrasound (B-mode), aiming at the general assessment of brain anatomy; color Doppler ultrasound, with the purpose of mapping the vascularization of the right and left middle cerebral artery (MCA) and the basilar artery (BA), and pulsed Doppler ultrasound to obtain wave spectra and calculate arterial hemodynamic indexes.

### Transcranial ultrasound examination

For the ultrasound examination, the animals were taken to the ultrasound room and placed on their tutors’ lap for the examination, initially with their heads towards the monitor (for left-side assessment) and their bodies parallel to the equipment, and later with their heads in the opposite direction to the monitor (for right-side assessment) keeping their bodies parallel to the equipment. The dogs were then gently restrained in the head region to prevent movement and the occurrence of artifacts during the sonographic examination. No trichotomy was necessary in any of the acoustic windows used for transcranial sonographic scanning since the application of water and gel was sufficient to visualize the structures of interest.

Transcranial ultrasound scans (B-mode and Doppler) were carried out on the MyLab^®^ X8 Platform (Esaote, Italy) using a low-frequency microconvex multifrequency transducer (3–11 MHz). In some puppies and miniature dogs, a high-frequency linear transducer (4–15 MHz) was also used to assess the structures during the sonographic scan. The image settings (gain, frequency, focus, TGC and depth) were preferably kept at constant values for better standardization of the technique and adapted according to physical characteristics and size of each dog. The power of the equipment was kept at 100% and the wall filter at 100 Hz to minimize possible artifacts from adjacent tissues or movement artifacts during the sonographic scan.

As for the Doppler settings, the sample volume was kept between 1 mm and 1.5 mm, with the minimum value used for the main cerebral arteries (small caliber) and the maximum value for the basilar artery (larger caliber). The scale or pulse repetition frequency (PRF) varied between 1.5 kHz and 4.5 kHz (depending on the artery assessed and the size of the dog) and the frequency was kept at 4.2 MHz. The insonation angle was adjusted to values greater than zero and less than 60°. The baseline and gain of the pulsed Doppler were also adjusted at the time the pulsed spectrum was acquired, with the aim of improving the spectral tracing and avoiding the occurrence of artifacts.

**Fig. 5:**
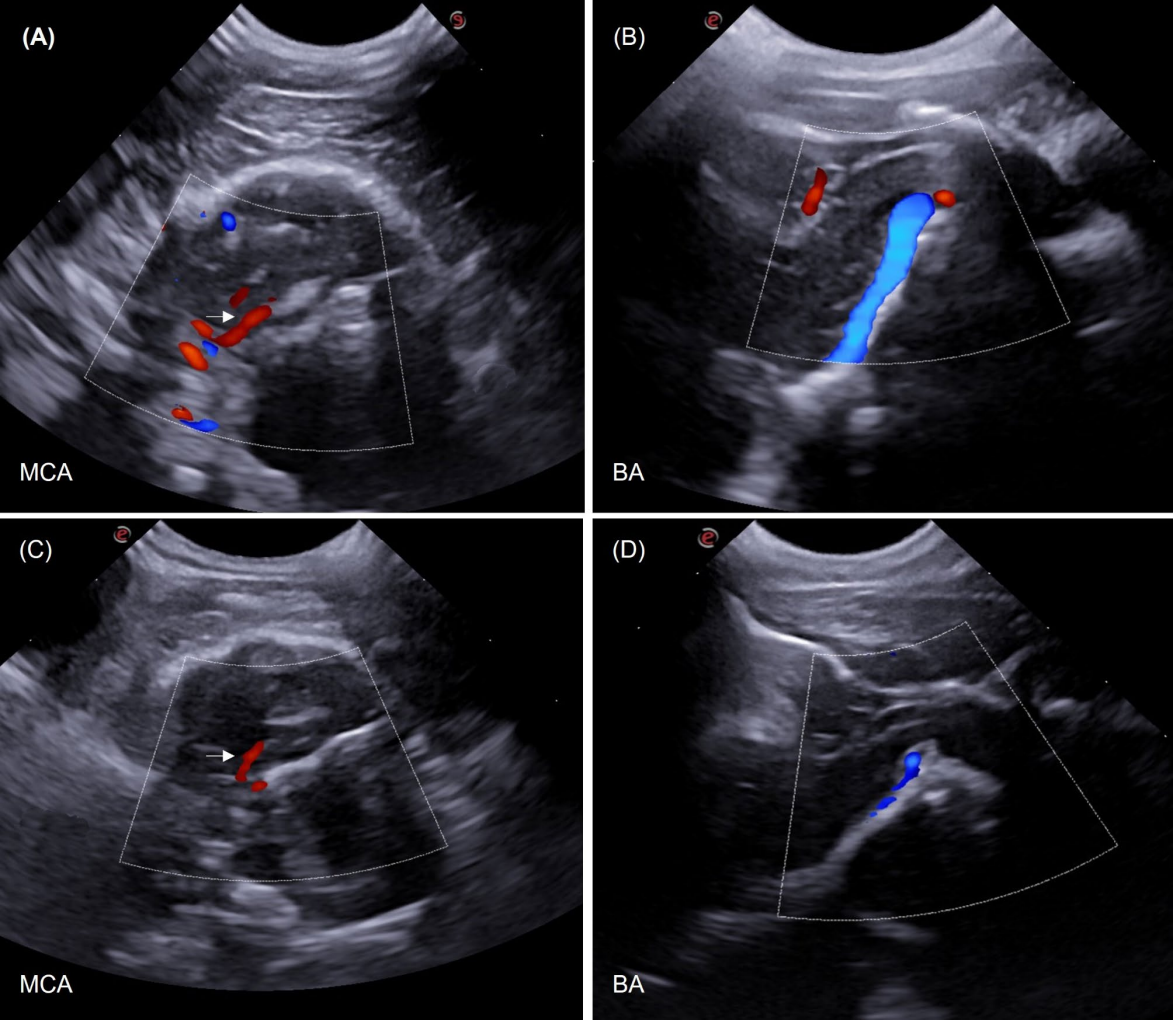
Transcranial sonographic images from the color Doppler study of the middle cerebral artery (MCA) and basilar artery (BA) of young, adult and elderly dogs. (**A**) and (**B**) MCA (arrow) and BA (blue flow) of puppies. (**C**) and (**D**) MCA (arrow) and BA of an elderly dog. Doppler adjustments (**A**) Color Doppler gain = 65% and PRF = 1.9 kHz; (**B**) Color Doppler gain = 66%; PRF = 3.5 kHz; (**C**) Color Doppler gain = 60%, PRF = 1.5 KHZ; (**D**) Color Doppler gain = 60%; PRF = 1.5 KHz

Initially, a B-mode assessment of the general encephalic anatomy was carried out according to protocols described in the literature [19, 20, 33], looking for any abnormalities that could lead to the animal being excluded. After the B-mode examination, Doppler ultrasound of the brain was performed to assess the MCA of both cerebral hemispheres and BA, making the necessary adjustments for hemodynamic assessment. Different windows used for performing TCDUS are shown in Fig. 5.

To assess the middle cerebral arteries, the transducer was initially positioned dorsally to the zygomatic arch in the left and right transtemporal windows and rotated 45° clockwise from its central axis, obtaining a caudal oblique dorsal section of the brain, and 45° anticlockwise to obtain a dorsal oblique cranial section for the Doppler scan. When necessary, angulations of approximately 10° were made (“fan scan”) to better visualize the vessels studied, identified by their corresponding path and topography [19, 31]. In dogs, the MCA can be sonographically identified on the laterodorsally surface of each hemisphere marked by the pseudosylvian fissure, from where it ascends, adjacent to the optic chiasm region.

**Fig. 6:**
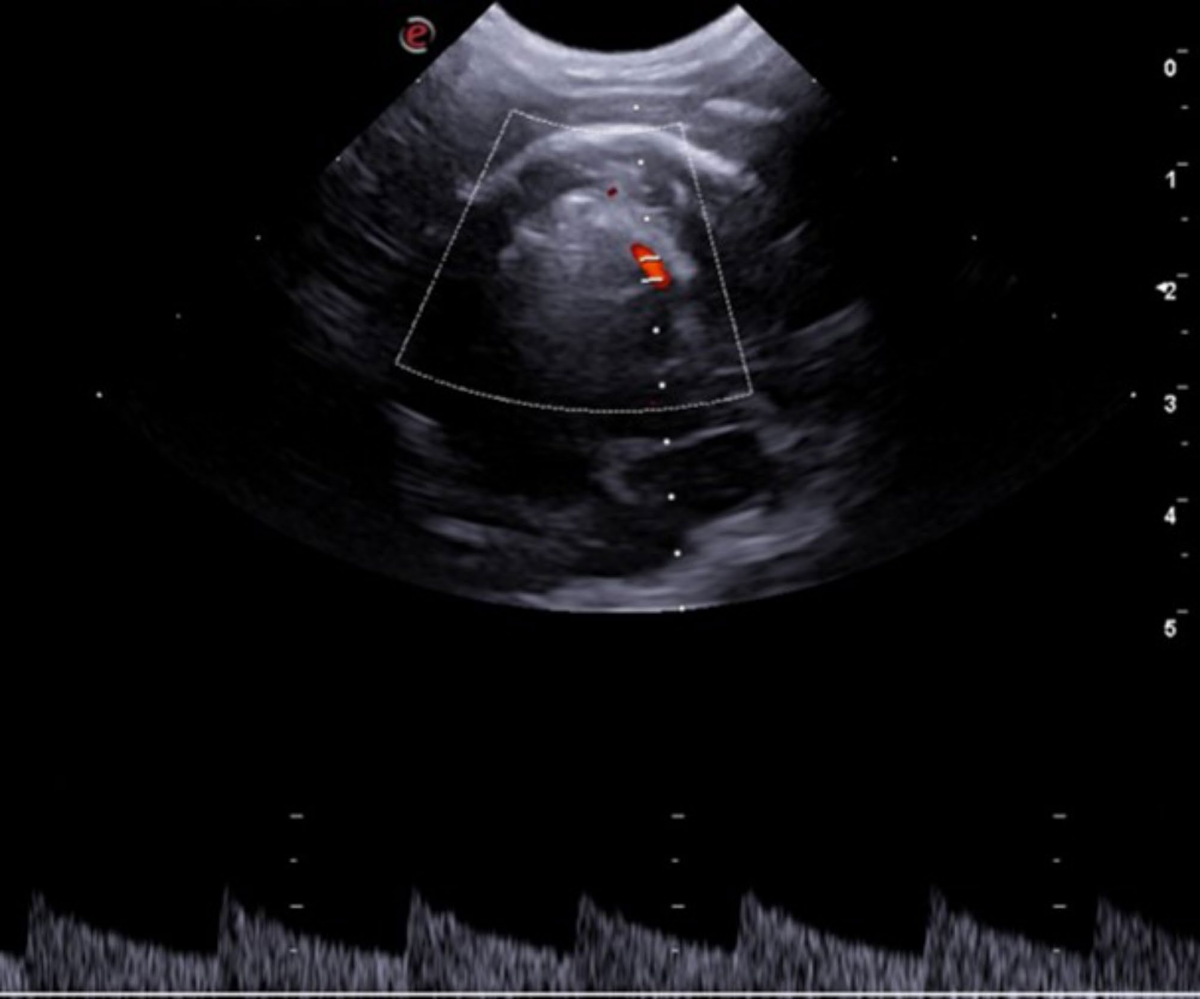
Sonographic image of normal morphology of the pulsed spectrum of the canine middle cerebral artery obtained in a temporal window (oblique dorsal plane), showing laminar flow with a low resistivity pattern with high diastolic flow. Doppler adjustments Color Doppler gain = 66%, PRF = 4.3) MHz

For the BA assessment, the scan was carried out through the suboccipital window, in transverse and sagittal planes, initially identifying the foramen magnum in B-mode and then activating the color Doppler for arterial identification. This artery can be easily identified sonographically by running along the midline of the ventral surface of the medulla and pons. For scanning, the patient’s neck was gently ventroflexed towards the sternum at an angle of approximately 30°, increasing the depth of the sample volume area and maximizing the Doppler signal. The Triplex Doppler mappings of the MCA and BA are shown in Fig. 6.

Once the vessels had been located, the pulsed Doppler was activated and a spectral trace with at least three similar sequential waveforms was collected. The graphs corresponding to blood flow were then analyzed in terms of their spectral tracing, using the nomenclatures proposed by Kim et al. (2020) [40].

When no artifacts were identified in the tracings, the vessels were assessed quantitatively by calculating the main hemodynamic indexes: pulsatility index (PI), resistivity index (RI), peak systolic velocity (PSV) and end-diastolic velocity (EDV). Regarding the Doppler ultrasound examination, the PSV and EDV values were measured in the proximal segment of the arteries under assessment. This was done to ensure consistency and capture the hemodynamic values at a point that reflects the general flow characteristics before branching or narrowing occurs.

**Fig. 7:**
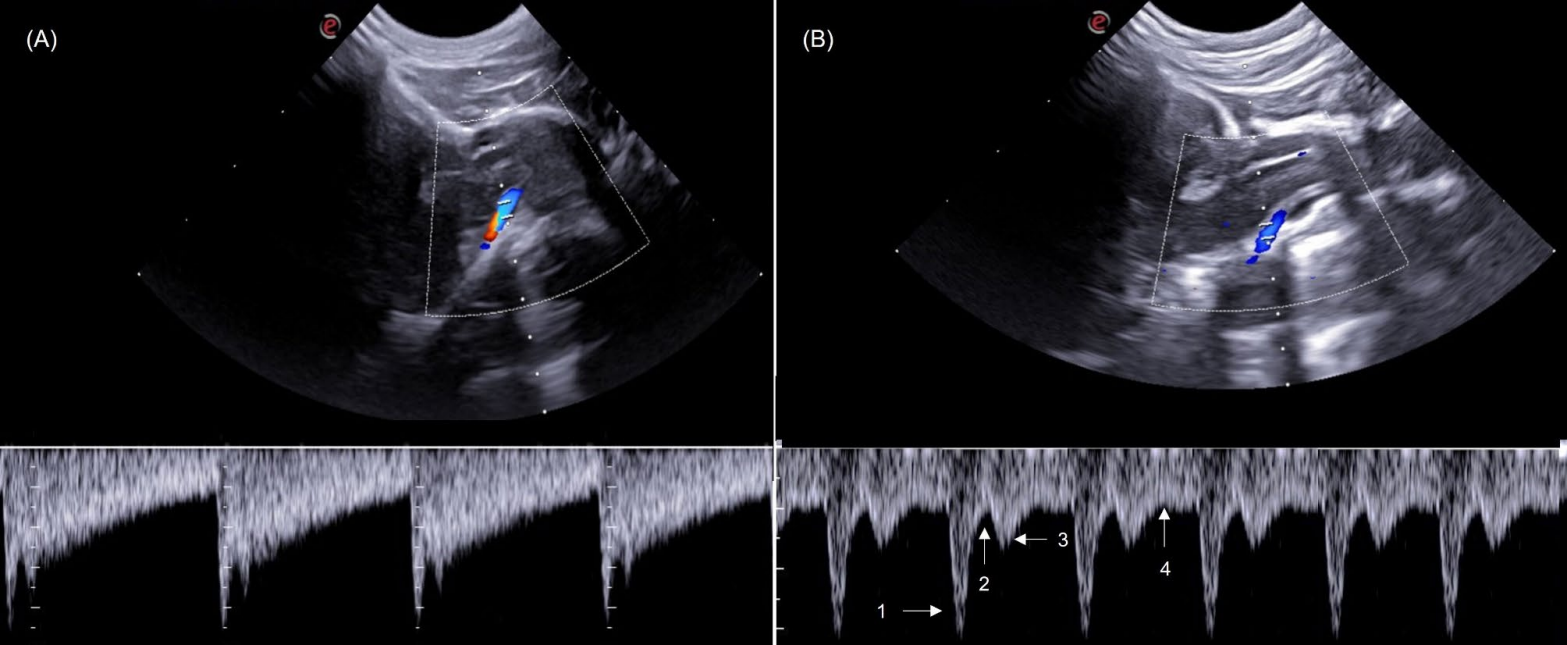
Sonographic images of transcranial pulsed Doppler examination of the basilar artery of canines showing pulsed Doppler mapping of the basilar artery obtained in the suboccipital window (longitudinal plane) of canines (**A**) pulsed spectrum showing normal morphology (**B**), pulsed spectrum with presence of dicrotic peaks, considered a variation of normality. 1 = Systolic peak; 2 = Dicrotic notch; 3 = Systolic point; 4 = Diastolic point

The operator manually traced three sequential waves that were as morphologically similar as possible to reduce the effects of physiological variation. The Doppler velocimetric parameters mentioned above were calculated electronically by the ultrasound machine´s software. For the manual trace, the caliper was positioned at the beginning of the systole of the first wave until the end of diastole of the last wave, resulting in a final average for each index studied (Fig. 7).

Manual rather than automatic tracing were chosen to minimize possible measurement errors, since it has been observed that automatic measurements can underestimate PSV and RI values and overestimate EDV values, thus impairing their reliability [42]. The values obtained were compared to those found to date in veterinary literature.

### Statistical analysis

Qualitative variables which were mainly binary (yes or no) were transformed into contingency tables and compared between age groups (percentages) using the Chi-squared test. Quantitative variables were initially assessed for normal distribution (Shapiro-Wilk test), then compared between age groups (young, adult, elderly) using the ANOVA test (if necessary, the data was normalized using the Lambda method) and if this was significant, Tukey’s post-test was applied to compare the means of the groups with each other; and between the sexes using the Student’s t-test. Finally, a correlation test (Pearson) was carried out between the variables evaluated. The significance level for all the tests was set at *p* < 0.05 and the data was presented as percentage or mean ± standard deviation (SD) and 95% confidence intervals (95% CI) for each of the variables.

## Data Availability

No datasets were generated or analysed during the current study.

## Abbreviations

BA: Basilar Artery
CT: Computed Tomography
EDV: End-Diastolic Velocity
MCA: Middle Cerebral Artery
PI: Pulsatility Index
PSV: Peak Systolic Velocity
RI: Resistivity Index
TCDUS: Transcranial Doppler Ultrasound
TUS: Transcranial Ultrasound
